# Changes in the activation and function of the ankle plantar flexor muscles due to gait retraining in chronic stroke survivors

**DOI:** 10.1186/1743-0003-10-12

**Published:** 2013-01-31

**Authors:** Brian A Knarr, Trisha M Kesar, Darcy S Reisman, Stuart A Binder-Macleod, Jill S Higginson

**Affiliations:** 1 Biomechanics and Movement Science, University of Delaware, Newark, DE, USA; 2 Department of Physical Therapy, University of Delaware, Newark, DE, USA; 3 Department of Mechanical Engineering, University of Delaware, Newark, DE, USA; 4 Department of Rehabilitation Medicine, Division of Physical Therapy, Emory University, Atlanta, GA, USA; 5 University of Delaware, 126 Spencer Lab, Newark, DE 19716, USA

**Keywords:** Gait, Stroke, Musculoskeletal simulation, Plantar flexors, Muscle function

## Abstract

**Background:**

A common goal of persons post-stroke is to regain community ambulation. The plantar flexor muscles play an important role in propulsion generation and swing initiation as previous musculoskeletal simulations have shown. The purpose of this study was to demonstrate that simulation results quantifying changes in plantar flexor activation and function in individuals post-stroke were consistent with (1) the purpose of an intervention designed to enhance plantar flexor function and (2) expected muscle function during gait based on previous literature.

**Methods:**

Three-dimensional, forward dynamic simulations were created to determine the changes in model activation and function of the paretic ankle plantar flexor muscles for eight patients post-stroke after a 12-weeks FastFES gait retraining program.

**Results:**

An median increase of 0.07 (Range [−0.01,0.22]) was seen in simulated activation averaged across all plantar flexors during the double support phase of gait from pre- to post-intervention. A concurrent increase in walking speed and plantar flexor induced forward center of mass acceleration by the plantar flexors was seen post-intervention for seven of the eight subject simulations. Additionally, post-training, the plantar flexors had an simulated increase in contribution to knee flexion acceleration during double support.

**Conclusions:**

For the first time, muscle-actuated musculoskeletal models were used to simulate the effect of a gait retraining intervention on post-stroke muscle model predicted activation and function. The simulations showed a new pattern of simulated activation for the plantar flexor muscles after training, suggesting that the subjects activated these muscles with more appropriate timing following the intervention. Functionally, simulations calculated that the plantar flexors provided greater contribution to knee flexion acceleration after training, which is important for increasing swing phase knee flexion and foot clearance.

## Background

3The degree of locomotor impairment post-stroke can vary greatly [1], but a majority of individuals post-stroke have decreased walking speed and abnormal gait kinematics [2]. These post-stroke gait impairments are a critical target of rehabilitation. The use of treadmills has gained popularity as an intervention for gait retraining post-stroke [3-6]. Recent studies have investigated combining treadmill walking with more targeted rehabilitation methods such as functional electrical stimulation (FES) [7]. In particular, impairment of the plantar flexors, typical of stroke gait, has been the focus of recent rehabilitation approaches [8,9] because of the importance of both foot clearance and forward propulsion in post-stroke gait function [10].

It has been shown that functional electrical stimulation of the plantar flexors during pre-swing, along with the paretic ankle dorsiflexors during swing, provided additional gait benefits including increased swing phase knee flexion, plantar flexion at toe-off, and forward propulsion [8]. Furthermore, combining fast treadmill walking and FES applied to the plantar and dorsiflexors for individuals post-stroke [6] resulted in greater anterior ground reaction force, trailing limb angle, and swing phase knee flexion for individuals post-stroke compared to either fast walking or FES alone. While these immediate effects of fast walking combined with FES are encouraging, the mechanisms underlying the effects are unclear.

Computer simulation studies have previously been used to demonstrate the function of individual muscles in healthy [11-13] and post-stroke gait [14]. In particular, pre-swing has been highlighted as an important phase of the gait cycle for forward propulsion and swing initiation in healthy walking [15]. During this phase, it has been shown that the soleus is the primary contributor to forward propulsion and the gastrocnemius is the primary contributor to swing initiation in healthy gait [11,12,15]. Most recently, simulation analyses identified decreased paretic soleus and gastrocnemius contributions to forward propulsion in an individual post-stroke compared to a healthy control [16] and have suggested that rehabilitation strategies that increase paretic forward propulsion and swing initiation in a population post-stroke have great potential to improve gait performance post-stroke [16]. Similar results were shown with two simulations representing populations post-stroke with average walking speeds of 0.55 and 0.92 m/s [17]. Computer simulations have not been performed on individuals post-stroke with a wider range of gait impairments. Perhaps more importantly, changes in muscle function after a gait retraining intervention have not been investigated using musculoskeletal modeling.

Due to the role of plantar flexors and leg extension [18] in generation of propulsion and subsequent maintenance of gait speed, as well as knee flexion velocity which allows for greater swing-phase knee flexion [19], we developed and tested a novel gait training program combining fast treadmill walking with plantar and dorsiflexor FES during gait (FastFES). One goal of the FastFES gait retraining program was to improve push off forces during pre-swing and foot clearance during swing, two common deficits seen in post-stroke gait, by delivering FES to ankle plantar flexors. The FastFES intervention produced improvements in a wide variety of gait-related outcome measures including forward propulsion, walking speed, walking endurance, and activity [20]. Using musculoskeletal simulations to identify the functions of specific muscles in response to an intervention such as FastFES can be a useful method to assess and enhance gait retraining.

This study used subject-specific musculoskeletal models to simulate the changes in activation and function of the ankle plantar flexor muscles in individuals post-stroke after a FastFES gait retraining program, as well as to identify relationships between simulation results and clinical gait variables. We developed forward dynamic gait simulations using gait data collected before and after 12-weeks of FastFES gait training. The purpose of this study was to demonstrate that simulation results were consistent with (1) the purpose of the intervention and (2) expected muscle function during gait based on previous literature. Additionally, we evaluated the ability of the model results to predict intervention outcomes by demonstrating correlations between our simulation results and clinically relevant outcome measures.

## Methods

Twelve individuals post-stroke (age 63 ± 8.6 years, 3 men, >6 months post-stroke) were recruited to participate in a 12-week FES gait retraining intervention, involving both plantar and dorsiflexor stimulation [20]. Inclusion criteria were defined as: 6 months after a stroke involving cerebral cortical regions, able to walk for 5 minutes at self-selected speed without a brace or assistive device, passive paretic ankle dorsiflexion range of motion to reach at least of 5^°^ plantar flexion with the knee flexed, and presence of deficits in walking function. Exclusion criteria were defined as: severe aphasia, substantial cognitive deficits, cerebellar involvement, or preexisting conditions affecting walking function [20].

### Subject training

Training consisted of four six minute bouts of treadmill walking at the subjects’ fastest possible speed with FES delivered to the paretic limb during the first, third, and fifth minutes of each bout. A fifth bout consisted of three minutes of treadmill walking with FES followed by three minutes of overground walking without FES. During the overground walking subjects were instructed to walk with the same pattern as practiced with the FES. This fifth bout is designed to help transfer the training to a more natural overground setting. FES was delivered to the ankle dorsiflexor muscles during the paretic swing phase and to the ankle plantar flexor muscles during the paretic pre-swing phase of gait [8]. Timing of the FES during the gait cycle was controlled by two compression closing foot switches (25-mm diameter MA-153, Motion Lab Systems Inc., Baton Rouge, LA) attached to the sole of the paretic limb shoe under the fifth metatarsal head (forefoot switch) and the other under the lateral portion of the heel (hindfoot switch). The FES system delivered stimulation to the ankle dorsiflexor muscles during the paretic swing phase of gait, while the paretic foot was off the ground. The paretic ankle plantar flexor muscles were stimulated from heel off to toe off of during paretic limb pre-swing, as indicated by the hindfoot and forefoot switches. Subjects walked without a brace or assistive device during all training sessions. This training was done 3x/week for 36 total training sessions. All subjects signed informed consent forms approved by the Human Subjects Review Board at the University of Delaware.

### Data collection

Kinematic and kinetic gait data were collected using an 8-camera motion capture system (Vicon MX, Los Angeles, CA; Motion Analysis, Santa Rosa, CA) as subjects walked at their self-selected walking speed on an instrumented split belt treadmill (AMTI, Watertown, MA; Bertec, Columbus, OH) pre- and post- intervention. The self-selected walking speed was determined during over ground walking using the average of three trials of the 6-meter walk test. Subjects walked without a brace or assistive device and no FES was delivered during these evaluation sessions. Subjects wore an overhead support harness with no body weight support and held on to a handrail (if needed) for safety.

Three-dimensional, subject-specific forward dynamic simulations were created from motion capture walking trials using OpenSim [21]. The model had 54 actuators, three degrees of freedom at the pelvis and hip joints, and one degree of freedom at the knee, ankle and toe joints, and was scaled to the subject’s size and mass. This exact model [22] and similar models [21,23] have previously been used in both healthy and osteoarthritic populations. Residual reduction analysis was run on the data to calculate residual forces that account for dynamic inconsistencies between the kinematic and kinetic data, such as the use of handrails. The model predicted muscle activations and forces required to reproduce the experimentally measured gait kinetics and kinematics were found using the Computed Muscle Control algorithm [24]. During computed muscle control, a combination of static optimization and proportional-derivative control is used to compute the muscle excitation levels necessary to drive the kinematics of the model towards the experimentally measured coordinates. The optimization function solved in this step minimizes the sum of two terms: the sum of the squared actuator controls and the weighted sum of the errors in desired acceleration.

### Data analysis

Based on the muscles targeted by the intervention, the paretic medial gastrocnemius (MG), soleus (SOL), and tibialis posterior (TP) muscles were analyzed in this study. All analysis in this manuscript is focused on the paretic limb. While the authors acknowledge the importance of all muscles, both ipsilateral and contralateral, in contributing to the variables of interest in this study, the plantar flexor muscles are being analyzed here due to the targeted nature of the intervention. Of the three plantar flexor muscles, the MG and SOL muscles were directly stimulated by FES. The TP was also studied, as changes in the TP would reflect changes in a muscle that serves a similar function but was not exposed to FES. For each muscle, the simulated activation ranged from 0 to 1, where 0 indicates no muscle activation and 1 indicates full muscle activation. We calculated the average simulated activation over a period of time, where 1.0 would be the maximum average activation, for the complete gait cycle and during double support. Double support in this study is defined as the period of double support on the paretic limb during pre-swing. Muscle force perturbations were used to determine individual muscle contributions to knee joint and center of mass (COM) accelerations [25]. For this analysis, an individual muscle’s force is perturbed by ± 1 N and simulated forward for 0.01 s interval to calculate the changes in the model’s joint angle and center of mass accelerations [25]. A foot-ground contact model with linear and torsional springs is used to account for the change in ground contact forces caused by the force perturbation. The induced accelerations were averaged over the double support phase of gait for each muscle. For each muscle, changes in percent activation post-intervention were tested for correlation with changes in peak knee flexion and induced knee flexion acceleration. The relationship between trailing limb angle and forward COM acceleration by the plantar flexor (PF) muscles was also examined. In addition, we examined the relationships between changes in percent activation of the PF muscles versus changes in peak knee flexion during swing phase of gait and walking speed and between forward COM acceleration produced by the PF muscles versus walking speed.

### Statistical analysis

Differences between simulated activation for each muscle pre- and post- intervention were evaluated using the Wilcoxon signed-rank test. Non-parametric statistics per performed due to low sample size and high variability between subjects. Changes in the average knee joint and COM accelerations from pre- to post-intervention were assessed on an individual basis. Linear regressions were performed to assess the following relationships: (1) Changes in simulated activation of the PF muscles versus changes in peak knee flexion during swing phase of gait and walking speed. (2) Changes in simulated plantar flexion activation and trailing limb angle versus changes in knee flexion acceleration. (3) Forward COM acceleration produced by the PF muscles versus walking speed.

## Results

Four subjects were excluded from this study due to inaccurate force plate data as a result of equipment failure. A total of 16 simulations were generated, with a pre-training and post-training simulation for each of the eight remaining subjects, built from the gait trials at self-selected speed at the time of collection. All subjects improved self-selected walking speed, and the self-selected speed post-intervention speed was faster (Median 0.2 m/s (Range [0.1, 0.6], *P* < 0.01)) than pre-intervention (Table 1).

**Table 1:** Subject characteristics at the pre-training evaluation

**Subject**	**Gender**	**Age**	**Side of**	**Time since**	**Self- selected**		**Fugl-Meyer**
			**Hemiparesis**	**Stroke**	**gait speed**		**(LE) Score**
		**(yrs)**	**(L/R)**	**(months)**	**(m/s)**		**Max = 34**
					***Pre***	***Post***	
**98**	M	66	R	19	0.30	0.40	21
**108**	M	70	L	21	0.50	0.60	13
**110**	F	65	R	15	0.30	0.90	18
**128**	F	65	R	18	0.50	0.70	18
**129**	F	54	R	55	0.50	0.80	17
**136**	F	58	R	12	0.30	0.50	13
**137**	M	46	R	8	0.40	0.50	15
**142**	F	70	L	9	0.30	0.50	22

Self-selected gait speed listed for pre- and post-intervention.

### Muscle activation during gait

**Figure 1:**
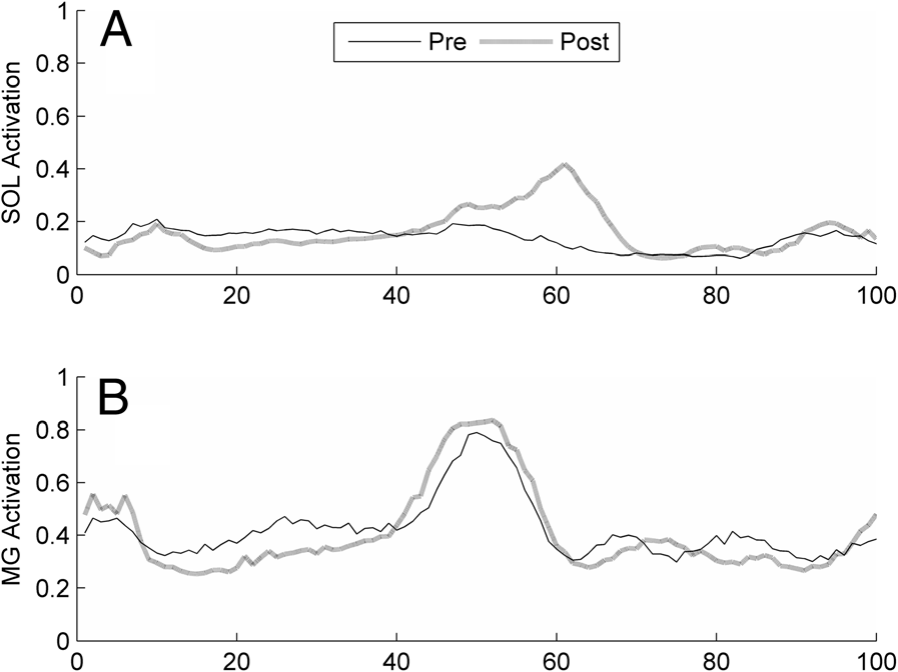
**Average activation of the paretic (a) soleus and (b) medial gastrocnemius, muscle over the full gait cycle pre- and post-intervention for one representative subject.** Y-axis indicates model activation, where 0 represents no activation and 1 represents full activation.

A median increase of 0.07 (Range [−0.01,0.22]) was seen in simulated activation averaged across all plantar flexor muscles over double support after 12-weeks of gait training (*P* = 0.016). Medial gastrocnemius experienced the largest median change across subjects, at 0.12 (Range [−0.03,0.19] ) (*P* = 0.04), and a 0.04 (Range [0.01, 0.11]) median increase (*P* = 0.01) was seen in the tibialis posterior muscle. Although not significant, the soleus muscle showed a median increase of 0.05 (Range [−0.07, 0.44]) with training (*P* = 0.20), with 5 of 8 subjects increasing predicted activation and two subjects showing no change (< 0.01). No significant change with training was seen for the average of these muscles over the full gait cycle (median 0.01 (Range [−0.06, 0.04])) (Figure 1).

### Muscle function during gait

#### Forward COM acceleration

**Figure 2:**
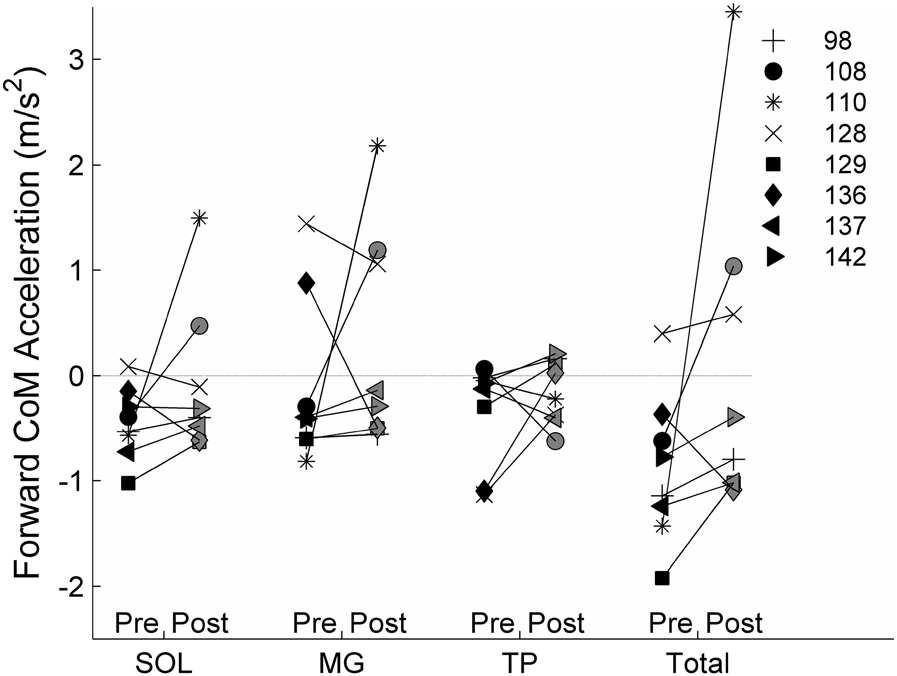
**Forward center of mass acceleration induced by the plantar flexor muscles during double support pre- and post-intervention.** More positive values represent increased contribution of muscles to forward acceleration.

Prior to training, the sum of the PF muscles for seven of the eight subjects decelerated the COM in the direction of walking during double support; only one subject’s PF showed the normal pattern of accelerating the COM forward during double support (Figure 2). Although the changes in plantar flexor function after training (Median 0.36 m/s^2^ (Range [−0.72, 4.89])) were not statistically significant for the total or individual muscles across subjects, two additional subjects (3 total) achieved forward COM acceleration from the PF muscles post-intervention. Overall, the sum of the PF muscles for seven of the eight subjects either improved acceleration of the COM or decelerated the COM less after training.

#### Knee joint acceleration

**Figure 3:**
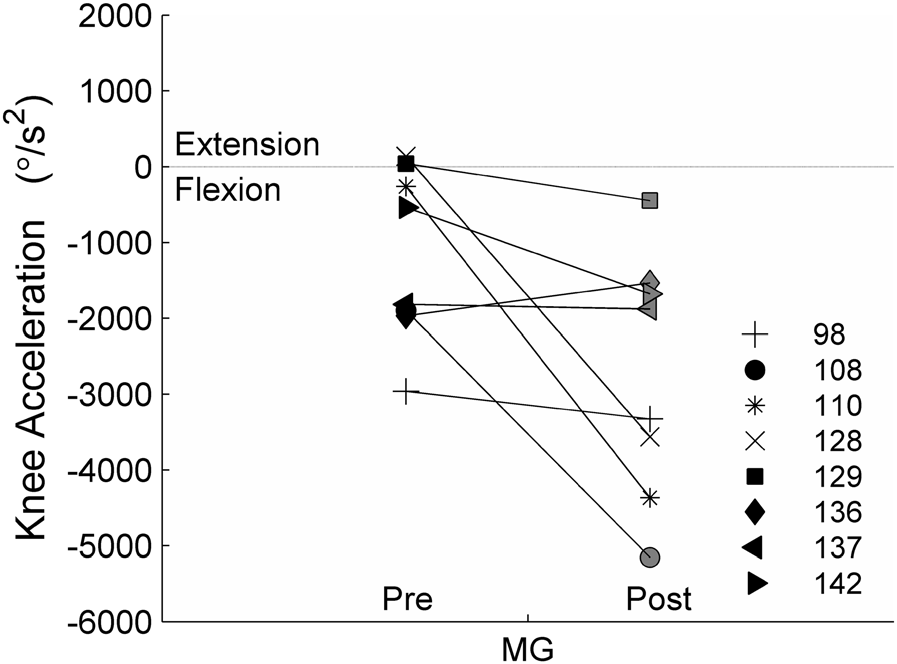
**Knee angular acceleration induced by the medial gastrocnemius during double support pre- and post-intervention.** More negative values represent greater knee flexion acceleration.

Overall, knee flexion acceleration induced by the medial gastrocnemius during double support increased significantly (Median −816.65 m/s^2^ (Range [−4108.4, 433.37])) (*P* = 0.04) post-intervention (Figure 3), with only one subject showing decreased induced flexion acceleration post-intervention. Prior to training, the medial gastrocnemius for two of the subjects induced extension acceleration at the knee during double support. In contrast, all subjects showed induced knee flexion acceleration from the medial gastrocnemius post-training.

### Clinical correlations

**Figure 4:**
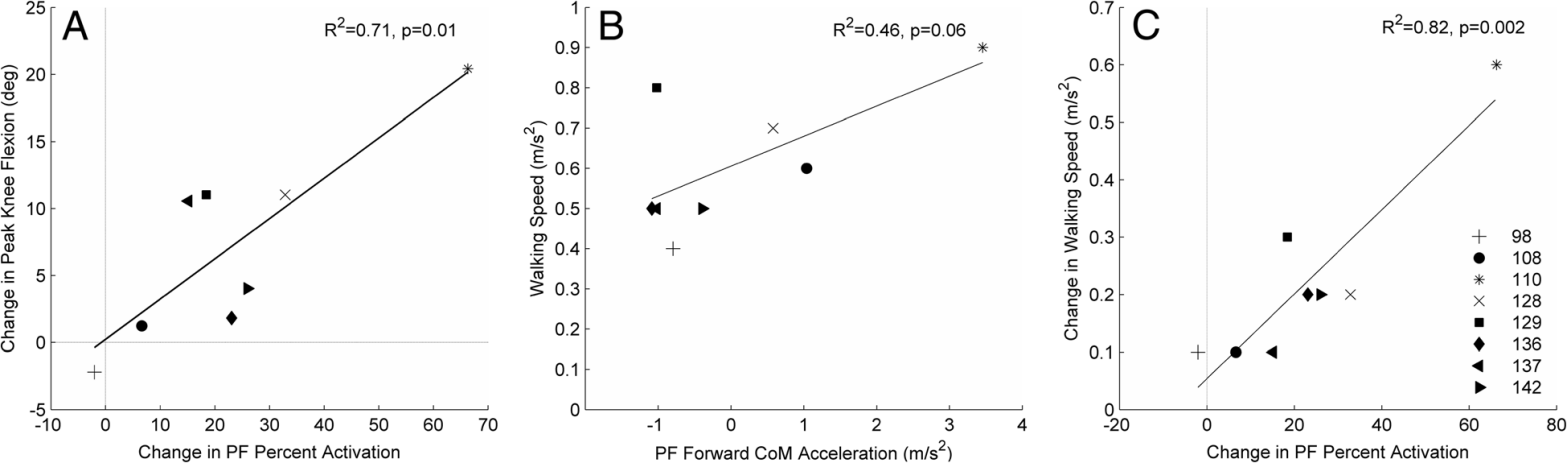
**Change in PF activation vs. peak knee flexion from pre- to post-intervention (A).** Post-training forward center of mass acceleration by the plantar flexors vs. post-training self-selected walking speed (**B**). Change in plantar flexor percent activation vs. change in self-selected walking speed from pre- to post-intervention (**C**).

Increases from pre- to post-training in model predicted PF activation during pre-swing showed a significant positive relationship (p = 0.01, R^2^ = 0.71) with increases in peak knee flexion during the swing phase of gait (Figure 4). A multiple linear regression with change in average simulated plantar flexion activation and trailing limb angle together showed a significant relationship to change in knee flexion acceleration induced by the plantar flexors during double support, explaining ~75% of the variance (p = 0.03, R^2^ = 0.75). Neither change in simulated PF activation nor trailing limb angle alone were significant predictors of change in knee flexion acceleration induced by the plantar flexors. Increased simulated plantar flexion activation with training (p = .002, R^2^ = .82) was related to increases in self-selected walking speed. Greater self-selected walking speed post-intervention exhibited a trend for a relationship with greater forward COM acceleration by the plantar flexors during double support (p = 0.06, R^2^ = .46).

## Discussion

The purpose of this study was to demonstrate that simulation results of plantar flexion activation and function for individuals pre- and post-intervention were consistent with (1) the purpose of the intervention and (2) expected muscle function during gait based on previous literature. In addition, we identified correlations between simulation results and clinical measures of walking function. Sixteen subject-specific models were created to represent pre- and post-training gait for 8 individuals post-stroke. To our knowledge, this is the first study to use musculoskeletal simulations of persons with chronic hemiparesis to simulate changes in muscle activation and function before and after participation in a gait retraining intervention.

Significant increases were seen in simulated plantar flexor activation during double support with no changes in total predicted activation over the entire gait cycle, suggesting selective increases in predicted plantar flexion activation during the phase of gait targeted by the intervention, i.e. double support. This new simulated activation pattern post-intervention is consistent with the timing of PF FES used during the intervention, which stimulated PF muscles only during pre-swing. This post-intervention activation pattern also agrees with healthy PF muscle coordination where the plantar flexors are activated most during late single leg stance and pre-swing. Thus, this new simulated pattern of activation for the plantar flexor muscles suggests that the subjects activated these muscles with more appropriate timing following the intervention.

The soleus and gastrocnemius play key roles in forward progression and swing initiation, respectively, primarily during the double support phase of gait [11]. Post-stroke muscle weakness of the PF muscles can greatly limit force generation by these muscles during gait, leading to decreased walking speed, limited swing phase knee flexion and poor foot clearance during swing [26,27]. Functional electrical stimulation during the FastFES intervention targeted the plantar flexor muscles pre-swing with the goal of enhancing the contribution of these muscles to their respective subtasks and improving gait.

In hemiparetic individuals, enhancing the contribution of the soleus towards forward COM acceleration has been identified as an important mechanism for increasing walking speed [16]. Interestingly, our models simulated that for 7 of 8 subjects post-stroke pre-training, the soleus decelerated the COM during double support, which is contrary to what is expected for healthy walking [11,28]. This suggests that the subjects’ paretic limb was in a poor biomechanical position for the soleus to accelerate the body forward. Post-training, the simulated PF muscles decelerated the COM less than pre-training for seven of eight subjects. In addition, simulations of 3 subjects began to accelerate the COM forward with the PF muscles post-intervention. Not surprisingly, these three subjects were three of the four fastest walkers post-training. Interestingly, the subject who showed the largest simulated increase in plantar flexor induced forward COM acceleration with training also showed the largest increase in self-selected walking speed (0.3 to 0.9 m/s), and walked at the fastest speed of the eight subjects post-intervention. It is important to note that the subjects in this study were walking at very slow speeds, ranging from 0.3 to 0.5 m/s pre-intervention, and the deceleration caused by the PF muscles pre-swing was likely a limiting factor to walking speed. A similar result was seen in a recent simulation study on a single individual post-stroke walking at a slightly faster speed of 0.6 m/s. [16] Peterson et al. highlighted forward propulsion as a limiting factor for walking speed, and also reported that the gastrocnemius contributes to negative acceleration of the pelvis during pre-swing [16], similar to the negative COM acceleration by the medial gastrocnemius found in our study.

Neptune and colleagues have shown that the gastrocnemius muscle is critical for swing initiation during pre-swing, increasing swing phase knee flexion and enabling foot clearance during swing by accelerating the knee into flexion [15]. For seven out of eight of our subjects, the simulated medial gastrocnemius exhibited an increased contribution to knee flexion acceleration post-intervention. This increase in knee flexion acceleration is associated with improved trailing limb posture combined with simulated activation of the gastrocnemius during double support, resulting in a change in muscle timing relative to joint posture. Additionally, the increase in simulated knee flexion acceleration was concurrent with increased walking speed and is consistent with previous simulation studies [15,29] that suggested that increased contribution to swing initiation by the gastrocnemius is required to increase walking speed. However, this is the first study to actually demonstrate that improved simulated plantar flexor activation is related to improved simulated knee flexion acceleration and walking speed, as was predicted by the previous cross-sectional analysis [15,29]. Moreover, the simulations allowed an analysis of the contribution of changes in plantar flexor activation to changes in knee flexion acceleration, something that cannot be examined through experimental data. This serves as an example of how muscle-actuated simulations can enhance our understanding of changes with intervention beyond what can be ascertained from experimental data alone.

In a previous experimental study investigating immediate effects of FES, peak knee flexion during swing was hypothesized to increase with greater forward propulsive forces as a result of increases in simulated PF activation during pre-swing [8]. In our simulations, PF activation correlated positively with peak knee flexion. This relationship was partially explained by gastrocnemius function (Figure 2), which can achieve increases in knee flexion directly through the bi-articulation at the ankle and knee, and by increased trailing limb angle. Leg extension by the trailing limb has been suggested to be important for achieving propulsion in persons with stroke [2,18] and was one goal of the fast treadmill training intervention used in this study. By using fast walking to increase the trailing limb angle, the ground reaction force exerted at push-off was directed in a more horizontal orientation, providing a greater propulsive force in the forward direction. This greater propulsive force probably allowed for increased forward acceleration of the body and limbs and enabled faster walking speeds and greater knee flexion during swing. Also, greater forward COM acceleration simulated by our model was predictive of greater self-selected walking speed post-training, a relationship that is consistent with previous cross-sectional study predictions [16].

This study examined changes in simulated post-stroke muscle activation and function at self-selected speed after a gait retraining intervention. Due to the limited number of subjects and the high variability in individuals post-stroke, it is not known if the findings of this study apply to the population of individuals post-stroke as a whole. Also, all subjects walked at a faster self-selected walking speed post-intervention. While the use of greater walking speeds post-intervention may have confounding effects on variables such as forward COM acceleration, we believe this analysis is important because it allows us to determine what changes in model predicted muscle activation and function were necessary to achieve the increases seen in walking speed post-intervention, a common goal of gait is retraining. In fact, this the first study to show concurrent improvements in speed, propulsion, and predicted PF activation after a targeted training intervention with individuals post-stroke. Due to the absence of a control group, it is not clear if the changes seen from pre- to post-intervention are a specific result of either FES or fast treadmill walking. Future work will include groups trained with FES or at fast walking speeds to assess the individual components of the intervention.

The analysis of muscle function is somewhat limited since activation was simulated without the use of EMG for this study. However, our modeling does account for the effect of limb posture on muscle function, which is not accounted for by EMG. For some of the muscles included in this model, surface EMG cannot be obtained. When used longitudinally with an intervention, EMG magnitude can be insensitive to hypertrophy of muscle, which makes comparisons difficult over time. Additionally, relying on EMG signal amplitudes can be difficult, as signal magnitude can vary based on electrode placement and tissue conductivity [30]. However, EMG can be useful for constraining timing of muscle activity, and future studies should consider its use. Additionally, the cost function used in the model minimizes the sum of the squares of the muscle activations, and generic muscle properties were used. Maximum isometric force parameters were not changed pre- to post-intervention, so it is possible that some of the increase in model predicted activation was due to strength increases elicited by the intervention. The selection of the cost function in particular could have an impact on the muscle activations predicted, as it is possible that the use of a different cost function could result in a different pattern of muscle activations which also reproduce the experimental kinematics and kinetics.

Subjects were allowed to use handrails during walking trials for safety purposes. Although the subjects were instructed to use the handrails with a ‘light touch,’ some subjects may have applied larger forces to the handrails. While the forces applied to the handrails were not accounted for explicitly, residual forces were calculated during the simulation and applied to the COM to account for kinetic imbalances due to external forces (i.e. handrail force). These residual forces were found to agree closely with the forces applied on the instrumented handrails during data collection.

## Conclusions

For the first time, muscle-actuated simulations were used to detect the effect of a gait retraining intervention on post-stroke modeled muscle activation and function. Improvements predicted by the simulations were consistent with improvements in kinematic measures of gait performance, demonstrating that musculoskeletal simulations can provide insight into clinically meaningful results. The simulations showed that a new pattern of predicted activation for the plantar flexor muscles emerged after training, suggesting that the subjects activated these muscles with more appropriate timing following the intervention. Functionally, after training, the plantar flexors provided greater contribution to knee flexion acceleration, which is important for increasing swing phase knee flexion and foot clearance. Improvement in trailing limb angle combined with increased predicted activation of the plantar flexors was shown to predict improvements in forward COM acceleration produced by the plantar flexors pre-swing. Also, greater contribution to forward COM acceleration by the plantar flexors after training was predictive of greater self-selected walking speed post-training. The correlations seen in this study are noteworthy, as they demonstrate a connection between musculoskeletal model predictions and clinically relevant gait variables measured pre- and post- intervention

## Abbreviations

FES: Functional electrical stimulation; PF: Plantar flexors; COM: Center of mass; MG: Medial gastrocnemius; SOL: Soleus; TP: Tibialis posterior; EMG: Electromyography.

## Competing interests

The authors declare that they have no competing interests.

## Authors’ contributions

BK created the musculoskeletal models, analyzed the data, and drafted the manuscript. TK participated in the design of the study and coordination and helped to draft the manuscript. DR conceived of the study, and participated in its design and coordination and helped to draft the manuscript. SBM conceived of the study, and participated in its design and coordination and helped to draft the manuscript. JS conceived of the study, and participated in its design and coordination and helped to draft the manuscript. All authors read and approved the final manuscript.

## References

[B1] Mulroy SJ Gronley J Weiss W Newsam C Perry J Use of cluster analysis for gait pattern classification of patients in the early and late recovery phases following stroke Gait Posture 2003 18 114 125 10.1016/S0966-6362(02)00165-0 12855307

[B2] Olney SJ Richards C Hemiparetic gait following stroke. Part I: characteristics Gait Posture 1996 4 136 148 10.1016/0966-6362(96)01063-6

[B3] Sullivan K Knowlton B Dobkin B Step training with body weight support: effect of treadmill speed and practice paradigms on poststroke locomotor recovery Arch Phys Med Rehabil 2002 83 683 691 10.1053/apmr.2002.32488 11994808

[B4] Barbeau H Optimal outcomes obtained with body-weight support combined with treadmill training in stroke subjects1 Arch Phys Med Rehabil 2003 84 1458 1465 10.1016/S0003-9993(03)00361-7 14586912

[B5] Ada L Dean C Hall JM Bampton J Crompton S A treadmill and overground walking program improves walking in persons residing in the community after stroke: a placebo-controlled, randomized trial Arch Phys Med Rehabil 2003 84 1486 1491 10.1016/S0003-9993(03)00349-6 14586916

[B6] Kesar TM Reisman DS Perumal R Jancosko AM Higginson JS Rudolph KS Binder-Macleod SA Combined effects of fast treadmill walking and functional electrical stimulation on post-stroke gait Gait Posture 2010 33 309 313 21183351 10.1016/j.gaitpost.2010.11.019 PMC3042540

[B7] Kottink AIR Oostendorp LJM Buurke JH Nene AV Hermens HJ IJzerman MJ The orthotic effect of functional electrical stimulation on the improvement of walking in stroke patients with a dropped foot: a systematic review Artif Organs 2004 28 577 586 10.1111/j.1525-1594.2004.07310.x 15153151

[B8] Kesar TM Perumal R Reisman DS Jancosko AM Rudolph KS Higginson JS Binder-Macleod SA Functional electrical stimulation of ankle plantar flexor and dorsiflexor muscles: effects on poststroke gait Stroke 2009 40 3821 3827 10.1161/STROKEAHA.109.560375 19834018 PMC2827197

[B9] Embrey DG Holtz SL Alon G Brandsma BA McCoy SW Functional electrical stimulation to dorsiflexors and plantar flexors during gait to improve walking in adults with chronic hemiplegia Arch Phys Med Rehabil 2010 91 687 696 10.1016/j.apmr.2009.12.024 20434604

[B10] Bowden MG Balasubramanian CK Neptune RR Kautz SA Anterior-posterior ground reaction forces as a measure of paretic leg contribution in hemiparetic walking Stroke 2006 37 872 876 10.1161/01.STR.0000204063.75779.8d 16456121

[B11] Neptune RR Kautz SA Zajac FE Contributions of the individual ankle plantar flexors to support, forward progression and swing initiation during walking J Biomech 2001 34 1387 1398 10.1016/S0021-9290(01)00105-1 11672713

[B12] Zajac FE Neptune RR Kautz SA Biomechanics and muscle coordination of human walking-part I: introduction to concepts, power transfer, dynamics and simulations Gait Posture 2002 16 215 232 10.1016/S0966-6362(02)00068-1 12443946

[B13] Anderson FC Pandy MG Individual muscle contributions to support in normal walking Gait and Posture 2003 17 159 169 10.1016/S0966-6362(02)00073-5 12633777

[B14] Higginson JS Zajac FE Neptune RR Kautz SA Delp SL Muscle contributions to support during gait in an individual with post-stroke hemiparesis J Biomech 2006 39 1769 1777 10.1016/j.jbiomech.2005.05.032 16046223

[B15] Neptune RR Sasaki K Kautz SA The effect of walking speed on muscle function and mechanical energetics Gait Posture 2008 28 135 143 10.1016/j.gaitpost.2007.11.004 18158246 PMC2409271

[B16] Peterson CL Hall AL Kautz SA Neptune RR Pre-swing deficits in forward propulsion, swing initiation and power generation by individual muscles during hemiparetic walking J Biomech 2010 43 2348 2355 10.1016/j.jbiomech.2010.04.027 20466377 PMC2922425

[B17] Hall AL Peterson CL Kautz SA Neptune RR Relationships between muscle contributions to walking subtasks and functional walking status in persons with post-stroke hemiparesis Clin Biomech 2011 26 509 515 10.1016/j.clinbiomech.2010.12.010 PMC3086953 21251738

[B18] Peterson CL Cheng J Kautz SA Neptune RR Leg extension is an important predictor of paretic leg propulsion in hemiparetic walking Gait Posture 2010 32 451 456 10.1016/j.gaitpost.2010.06.014 20656492 PMC2974765

[B19] Goldberg SR Anderson FC Pandy MG Delp SL Muscles that influence knee flexion velocity in double support: implications for stiff-knee gait J Biomech 2004 37 1189 1196 10.1016/j.jbiomech.2003.12.005 15212924

[B20] Kesar T Plantar- and Dorsi-Flexor FES in Conjunction with Fast Treadmill Training: Effects on Post-Stroke Walking Patterns12th Annual Conference of the International Functional Electrical Stimulation Society 2007 Philadelphia, PA, USA: Shriners Hospital for Children 4

[B21] Delp SL Anderson FC Arnold AS Loan P Habib A John CT Guendelman E Thelen DG OpenSim: open-source software to create and analyze dynamic simulations of movement IEEE Trans Biomed Eng 2007 54 1940 1950 18018689 10.1109/TBME.2007.901024

[B22] Richards C Higginson JS Knee contact force in subjects with symmetrical OA grades: differences between OA severities J Biomech 2010 43 2595 2600 10.1016/j.jbiomech.2010.05.006 20627301 PMC2937066

[B23] Xiao M Higginson JS Muscle function may depend on model selection in forward simulation of normal walking J Biomech 2008 41 3236 3242 10.1016/j.jbiomech.2008.08.008 18804767 PMC2586943

[B24] Thelen DG Anderson FC Using computed muscle control to generate forward dynamic simulations of human walking from experimental data J Biomech 2006 39 1107 1115 10.1016/j.jbiomech.2005.02.010 16023125

[B25] Liu MQ Anderson FC Pandy MG Delp SL Muscles that support the body also modulate forward progression during walking J Biomech 2006 39 2623 2630 10.1016/j.jbiomech.2005.08.017 16216251

[B26] Nadeau S Gravel D Arsenault AB Bourbonnais D Plantar flexor weakness as a limiting factor of gait speed in stroke subjects and the compensating role of hip flexors Clin Biomech 1999 14 125 135 10.1016/S0268-0033(98)00062-X 10619100

[B27] Parvataneni K Olney SJ Brouwer B Changes in muscle group work associated with changes in gait speed of persons with stroke Clin Biomech 2007 22 813 820 10.1016/j.clinbiomech.2007.03.006 17512646

[B28] McGowan C Neptune RR Independent effects of weight and mass on plantar flexor activity during walking: implications for their contributions to body support and forward propulsion J Appl Physiol 2008 78712 486 494 18556431 10.1152/japplphysiol.90448.2008 PMC2519947

[B29] Neptune RR Zajac FE Kautz SA Muscle force redistributes segmental power for body progression during walking Gait and Posture 2004 19 194 205 10.1016/S0966-6362(03)00062-6 15013508

[B30] De Luca CJ The use of surface electromyography in biomechanics J Appl Biomech 1997 13 135 163

